# Measuring the crowd within again: a pre-registered replication study

**DOI:** 10.3389/fpsyg.2014.00786

**Published:** 2014-07-28

**Authors:** Sara Steegen, Laura Dewitte, Francis Tuerlinckx, Wolf Vanpaemel

**Affiliations:** Research Group of Quantitative Psychology and Individual Differences, Faculty of Psychology and Educational Sciences, University of LeuvenLeuven, Belgium

**Keywords:** crowd within, registered replication study, power analysis, wisdom of the crowd, effect size

## Abstract

According to the crowd within effect, the average of two estimates from one person tends to be more accurate than a single estimate of that person. The effect implies that the well documented wisdom of the crowd effect—the crowd's average estimate tends to be more accurate than the individual estimates—can be obtained within a single individual. In this paper, we performed a high-powered, pre-registered replication study of the original experiment. Our replication results are evaluated with the traditional null hypothesis significance testing approach, as well as with effect sizes and their confidence intervals. We adopted a co-pilot approach, in the sense that all analyses were performed independently by two researchers using different analysis software. Moreover, we report Bayes factors for all tests. We successfully replicated the crowd within effect, both when the second guess was made immediately after the first guess, as well as when it was made 3 weeks later. The experimental protocol, the raw data, the post-processed data and the analysis code are available online.

## 1. Introduction

Quantitative judgements to general knowledge questions are famously known to be more accurate when estimates are averaged over a crowd compared to the individual estimates (Surowiecki, 2004). When individuals are guessing independently of each other, the crowd's estimate will be closer to the truth than the majority of the individual guesses. This “wisdom of the crowds” effect has been observed in a wide range of applications, including weight guessing, ordering tasks and market predictions (e.g., Galton, 1907; Steyvers et al., 2009; Dani et al., 2012).

In an elegant experiment, Vul and Pashler (2008) showed that the wisdom of crowds can also be obtained within a single individual. Participants were asked to make their best guess on eight general knowledge questions. Immediately after completing this, participants were unexpectedly asked to make a second, different guess for each question. The results showed that, overall, the average of two estimates from one person was more accurate than a single estimate of that person. It thus seems as if people possess a crowd within they can consult.

Vul and Pashler (2008) further showed that increasing the independence between both guesses strengthens the crowd within effect. In particular, a second group of participants was asked to answer the same questions 3 weeks later instead of immediately after completing the first questionnaire. The benefit of averaging two guesses within a person was larger in this *delayed* condition than when the second guess was elicited immediately (i.e., the *immediate* condition).

On a practical level, the phenomenon that averaging multiple guesses within a person improves estimation accuracy has useful implications in daily life with respect to decision making, as it shows that judgements can benefit from the proverbial “sleeping on it.” The crowd within effect has also important theoretical implications, as it suggests that our knowledge is represented in internal probability distributions from which responses are sampled. As such, it provides a solid ground of evidence for the emerging idea that human reasoning rests on Bayesian inference (e.g., Tenenbaum et al., 2006; Jones and Love, 2011). The practical and theoretical appeal of the crowd within has resulted in extensive media coverage (e.g., Herbert, 2008; “The Crowd Within,” 2008) and 78 citations (according to Google Scholar, June 7, 2013).

We know of four studies that attempted to replicate the crowd within effect in the immediate condition, with mixed results. Two of these studies report the finding that averaging two successive guesses from one person provides better estimates than the single guesses (Hourihan and Benjamin, 2010; Rauhut and Lorenz, 2011)^1^. The results from the two remaining studies were somewhat mixed. In line with the crowd within effect, Herzog and Hertwig (2009) showed that aggregating two guesses improved estimation accuracy compared to single guesses. However, the 95% confidence interval for this accuracy gain included the null value^2^. Finally, Edward Vul informed us about an unpublished replication attempt that failed to find a significant improvement of the average of two guesses compared to the first guess (Banker and McCoy, unpublished data). However, in support of the crowd within, the results did point in the expected direction (see Table 1 for more details).

**Table 1:** **Statistics for guess 1 and guess 2 in the immediate condition for the three studies included in the power analysis**.

**Study**	**Mean MSE**	**Mean MSE**	***SD* MSE**	***SD* MSE**	***r***	***n***	***t***	***p***	**${\hat{d}}_{z}$**
	**single guess**	**average guess**	**single guess**	**average guess**					
**GUESS 1**									
Vul and Pashler, 2008	555^*^	508^*^	361^*^	305^*^	0.88^*^	255^▵^	4.41^*^	<0.001^*^	0.28
Hourihan and Benjamin, 2010	502^▵^	484^▵^	261^◦^	268^◦^	0.91^◦^	170^▵^	2.15^▵^	0.03^▵^	0.16
Banker and McCoy, unpublished data	463^*^	452^*^	281^*^	268^*^	0.95^*^	201^▵^	1.71^*^	0.09^*^	0.12
**GUESS 2**									
Vul and Pashler, 2008	638^*^	508^*^	382^*^	305^*^	0.83^*^	255^▵^	9.90^*^	<0.001^*^	0.62
Hourihan and Benjamin, 2010	565^▵^	484^▵^	274^◦^	268^◦^	0.87^◦^	170^▵^	7.89^▵^	<0.001^▵^	0.61
Banker and McCoy, unpublished data	509^*^	452^*^	298^*^	268^*^	0.92^*^	201^▵^	7.03^*^	<0.001^*^	0.50

*The reported *t*-values of Vul and Pashler (2008) differ from what is reported in the original paper. Contacting the authors concerning the experiment led them to uncover that the data show a stronger evidence for the crowd within effect than what had been reported in the article originally. The *t*-statistic values for the crowd within effect on both the first and the second guess were reported smaller than they actually are. The reported t-values here are the correct ones*.

*MSE = mean squared error; SD = standard deviation; r = correlation between single guess and average guess; *n* = sample size; *t* = dependent *t*-statistic value; p = p-value of corresponding statistic; ${\hat{d}}_{z}$ = effect size*.

* Computed from raw data,

▵ numerically reported in paper,

◦ *derived from numerically reported statistics in paper*.

To the best of our knowledge, there are no replication attempts of the delayed condition. This condition yielded the strongest effect of the crowd within, which is in line with the idea that the benefit of averaging is a result of the different guesses being sampled from a probability distribution. Since a certain level of independence between the errors of the estimates is crucial in order to get this effect, it is logical that a three-week delay between the guesses (inducing a greater independence) enhances the benefit. Although this manipulation has never been adopted in other research studying the crowd within, a few studies did show that other factors boosting independence between the two guesses enhance the benefit of averaging (e.g., Herzog and Hertwig, 2009; Hourihan and Benjamin, 2010).

In the light of the practical and theoretical appeal of the crowd within, and the limited success in replicating the effect, we believe it is worthwhile to set up another attempt at replicating the crowd within effect in both the immediate and the delayed condition of Vul and Pashler (2008).

## 2. Method

Before the start of the data collection, we registered this study at the Open Science Framework (osf.io/p2qfv; Spies et al., 2012).

### 2.1. Sampling plan

#### 2.1.1. Immediate condition

The sampling plan for the immediate condition was based on a power analysis considering the existing evidence for the crowd within effect in this condition from the original paper by Vul and Pashler (2008) on the one hand, and from two replication attempts on the other hand, namely the study by Hourihan and Benjamin (2010) and the study by Banker and McCoy (unpublished data). The results from Herzog and Hertwig (2009) were not included in the power analysis, because these authors measured accuracy gain in a different way than Vul and Pashler (2008)^3^, making the reported statistics in these two studies incomparable. Finally, the results from Rauhut and Lorenz (2011) could not be considered, since this study did not report sufficient information to calculate the required effect sizes.

The power calculations were based on a weighted average effect size across the three relevant studies. As a measure of effect size, we used Cohen's standardized mean difference *d*_*z*_ for dependent groups (Cohen, 1988, p. 48):

(1) $$d_{z}=\frac{\mu_{X}-\mu_{Y}}{\sigma_{X-Y}}=\frac{\mu_{X}-\mu_{Y}}{\sqrt{\sigma_{X}^{2}+\sigma_{Y}^{2}-2\sigma_{X}\sigma_{Y}\rho_{XY}}},$$

where μ_*X*_ and μ_*Y*_ are the means in the two groups, σ_*X*_ and σ_*Y*_ are the standard deviations in the two groups and ρ_*XY*_ is the correlation between the pairs of observations. This effect size can easily be estimated from the *t*-statistic for dependent groups for a given effect and the corresponding sample size *n*, as follows:

(2) $${\hat{d}}_{z}=\frac{t}{\sqrt{n}}.$$

Table 1 shows the means, standard deviations, correlations between the pairs of observations, sample sizes, *t*-statistics and *p*-values of the immediate condition in all three studies, as well as the resulting effect sizes. Since the crowd within effect comprises an accuracy gain of the average guess compared to either of both single guesses, two effect sizes were calculated in each study: One effect size for guess 1 (i.e., the standardized mean difference between the mean squared error (MSE) of guess 1 and the MSE of the aggregated guess) and another effect size for guess 2 (i.e., the standardized mean difference between the MSE of guess 2 and the MSE of the aggregated guess).

We pooled the individual effect sizes across the studies by weighing each effect size with its inverse variance (Cooper et al., 2009):

(3) $$\bar{d_{z}}=\frac{\sum_{i\;=\;1}^{k}w_{i}{\hat{d}}_{z_{i}}}{\sum_{i\;=\;1}^{k}w_{i}},$$

where *k* is the number of studies and *w*_*i*_ is the inverse of the variance *v*_*i*_ of effect size ${\hat{d}}_{z_{i}}$ in study *i*:

(4) $$\frac{1}{w_{i}}=v_{i}=\left(\frac{1}{n_{i}}+\frac{{{\hat{d}}_{z_{i}}}^{2}}{2n_{i}}\right)2(1-r_{i}),$$

where *n*_*i*_ is the sample size and *r*_*i*_ is the correlation between the pairs of observations in study *i*.

Pooling the effect sizes across the three studies resulted in a weighted average effect size of *d*_*z*_ = 0.17 for guess 1 and *d*_*z*_ = 0.56 for guess 2.

Using G^*^Power 3.1 (Faul et al., 2009), we calculated a planned sample size for achieving a 0.95 power level using a two-tailed dependent *t*-test, given both effect sizes. This resulted in a sample size *n* = 439 for guess 1 and *n* = 31 for guess 2. To be most conservative, we planned to adopt at least the largest sample size of these two, that is *n* = 439.

#### 2.1.2. Delayed condition

As there are no known replication attempts of the crowd within effect in the delayed condition, the sampling plan for this condition was based on an effect size estimated from Vul and Pashler (2008) only. Again, we used Cohen's *d*_*z*_ as a measure of effect size, and we estimated an effect size for guess 1 and guess 2, using formula (2). The estimated effect sizes, as well as the means, standard deviations, correlations, sample sizes, *t*-statistics and *p*-values are shown in Table 2. Using G^*^Power 3.1, we calculated a planned sample size *n* = 48 for guess 1 and *n* = 13 for guess 2 in order to achieve a power of 0.95, using a two-tailed dependent *t*-test. To be conservative, we planned to adopt a sample size of at least *n* = 48 in the delayed condition.

**Table 2:** **Statistics for guess 1 and guess 2 in the delayed condition for the study included in the power analysis**.

**Study**	**Mean MSE**	**Mean MSE**	***SD* MSE**	***SD* MSE**	***r***	***n***	***t***	***p***	**${\hat{d}}_{z}$**
	**single guess**	**average guess**	**single guess**	**average guess**					
**GUESS 1**									
Vul and Pashler, 2008	542^*^	447^*^	363^*^	273^*^	0.84^*^	173^▵^	6.22^*^	<0.001^*^	0.47
**GUESS 2**									
Vul and Pashler, 2008	610^*^	447^*^	380^*^	273^*^	0.83^*^	173^▵^	9.85^*^	<0.001^*^	0.75

*The reported *t*-values of Vul and Pashler (2008) differ from what is reported in the original paper. See Table 1 for further information*.

*MSE = mean squared error; SD = standard deviation; *r* = correlation between single guess and average guess; *n* = sample size; *t* = dependent *t*-statistic value; p = p-value of corresponding statistic; ${\hat{d}}_{z}$ = effect size*.

* Computed from raw data,

▵ numerically reported in paper,

*◦ derived from numerically reported statistics in paper*.

#### 2.1.3. Recruitment

We recruited participants at the University of Leuven. Psychology students were asked to participate in the experiment either in turn for course credits (immediate condition) or for a chance to win cinema tickets (delayed condition). Given the characteristics of psychology students, we expected a majority of female participants between the age of 18 and 23. Following the original paper, participants did not have to meet any inclusion criteria. As will become clear below, we did not know beforehand the exact number of participants, so the sample sizes computed above are minimal sample sizes. Data were only analyzed once to avoid multiple comparison issues.

For the immediate condition, we recruited participants until we had reached at least the planned sample size of 439. In particular, we made use of sessions where participants were assigned in batches. The size of each batch was largely beyond our control, making the exact sample size unknown beforehand. We used the minimum number of batches to reach the planned minimal sample size of 439. The actual sample size was expected to be larger than 439.

For the delayed condition, we made use of a pool of about 300 students attending a course. All of them were invited to participate in an experiment consisting of two sessions. Three weeks after the first session, students who participated in the first session were invited to participate in the second session. Data were used from students who participated in both sessions (i.e., data from students who only participated in the first session were discarded). Again, the actual sample size was unknown beforehand, and was expected to be larger than 48^4^.

### 2.2. Materials and procedure

#### 2.2.1. Materials

The original material in the study of Vul and Pashler consisted of eight real-world knowledge questions^5^, shown in Table 3. We adopted these questions with updated answers (derived from *The World Factbook*; Central Intelligence Agency, 2013), as shown in Table 3. These questions were translated in Dutch.

**Table 3:** **Questions used in Vul and Pashler (2008) with original answers and updated answers**.

**No**.	**Question**	**Answers in Vul and Pashler (2008)**	**Answers in current study**
1	The area of the USA is what percent of the area of the Pacific Ocean?	6.3	6.3
2	What percent of the world's population lives in either China, India, or the European Union?	44.4	43.3
3	What percent of the world's airports are in the United States?	30.3	32.3
4	What percent of the world's roads are in India?	10.5	13.4
5	What percent of the world's countries have a higher fertility rate than the United states?	58	53.6
6	What percent of the world's telephone lines are in China, USA, or the European Union?	72.4	54.8
7	Saudi Arabia consumes what percentage of the oil it produces?	18.9	26.4
8	What percentage of the world's countries have a higher life expectancy than the United States?	20.3	22.4

#### 2.2.2. Procedure immediate condition

Participants were seated in front of computer screens in groups of 10–15 persons. At the beginning of the study, they were asked to activate full screen mode on their computer and to stay in this mode during the entire experiment. This was to prevent them from looking up the answers to the questions. Next, after signing an informed consent and providing their demographic details (sex, age, and nationality), participants were given the eight general knowledge questions sequentially, with the instruction to guess the correct answers and to not look them up. In line with the original study (Edward Vul, personal communication, June 6, 2013), the eight questions were presented in randomized order and participants were prevented from going back to earlier questions. Immediately after completing the first questionnaire, participants were unexpectedly asked to make a second, different guess for each question. Again, the questions were presented in randomized order. After completion, participants were asked to indicate whether they had looked up the answers to the questions or not.

#### 2.2.3. Procedure delayed condition

Students attending a course were asked to participate in a short experiment later that day, on the internet, without explaining the task they would have to perform. At the time of the experiment, participants were asked to activate full screen mode on their computer and to stay in this mode during the entire experiment. They were also asked not to seek help from anyone while performing the task. In line with the original study (Edward Vul, personal communication, October 19, 2013), participants were informed that there would be a second session of the experiment 3 weeks later, but without giving advance notice that they would be answering the questions a second time. After signing an informed consent and providing their demographic details (sex, age, and nationality), participants were given the eight general knowledge questions in the same way as in the immediate condition. After completing the questionnaire, participants were asked not to discuss the task with their companion students or other people, nor to look up the answers to the questions. Three weeks later, participants who participated in the first session of the experiment were invited per mail to participate in the second session, also on the internet. In this session, they were asked to give a second, different guess to each of the eight questions. At the end of the second session, participants were asked to indicate whether they had looked up the answers to the questions (either during the first session, during the second session or in the period between the two sessions) or not.

### 2.3. Known differences from original study

The study differed from the original study in two aspects. Firstly, as we explained above, the real-world knowledge questions used in the study of Vul and Pashler (2008) were translated to Dutch and the answers to these questions were updated.

Secondly, we expected that our subject pool of undergraduate students would be less diverse than the internet-based subject pool in the original study with respect to variables such as age, ethnicity and educational level. However, we do not believe this is critical for a fair replication, since there is no a priori or theoretical reason why the crowd within effect would rely on these type of variables.

### 2.4. Confirmatory analysis plan

#### 2.4.1. Data cleaning plan

Vul and Pashler (2008) did not adopt any data filtering procedures in their study (Edward Vul, personal communication, April 30, 2013). However, following the original authors' advice, we planned to exclude data from those participants which defocused the browser window running the study, as the latter may be an indication of participants looking up the answers to the questions. On the same line of reasoning, we planned to exclude data from participants who indicated that they had looked up the answers to the questions at the end of the experiment. Further, we planned to exclude data when impossible answers (i.e., percentages below zero or above hundred) or blank answers were given. In this case, both guesses for the concerning question were planned to be excluded from the analyses.

#### 2.4.2. Analysis process

A complete replication of the Vul and Pashler (2008) paper includes a higher accuracy of the aggregated guess compared to the individual guesses, in both the immediate and the delayed condition.

In accordance with the original study, we assessed for each participant the accuracy of a guess with the MSE of the estimate across all eight questions. In each condition, the MSE of guess 1, guess 2, and the average of both guesses was calculated for each participant. The MSE of the average was calculated by first averaging guess 1 and guess 2, and then computing the MSE. Next, we compared the MSE of guess 1 and the MSE of the average of both guesses across participants by performing a two tailed *t*-test for paired observations. Further, we repeated this for the comparison of the MSE of guess 2 and the MSE of the average of both guesses. For each condition, if the observed values of both *t*-statistics were positive (i.e., both the MSE of guess 1 and the MSE of guess 2 were on average larger than the MSE of the aggregated guess) and a *p*-value smaller than 0.05 was obtained for both the tests, we evaluated the replication of the crowd within effect as being successful in the concerning condition.

Besides these traditional metrics for evaluating the success of a replication attempt, we calculated the effect sizes, ${\hat{d}}_{z}$, for guess 1 (i.e., the standardized mean difference between the MSE of guess 1 and the MSE of the aggregated guess) and for guess 2 (i.e., the standardized mean difference between the MSE of guess 2 and the MSE of the aggregated guess), together with their 95% confidence intervals. This allowed us to consider subtleties in the replication outcomes beyond the traditional dichotomy of failure or success of the replication attempt (see Simonsohn, 2013).

Following Vul and Pashler (2008), we performed two additional tests. First, we also compared the difference in accuracy between guess 1 and guess 2, again by performing a two tailed *t*-test for paired observations. Vul and Pashler (2008) found that second guesses were less accurate than first guesses, indicating that the accuracy gain of averaging could not be attributed to subjects looking up the answers between guesses. Second, we compared the benefit of averaging in the immediate condition and the delayed condition by performing an unpaired *t*-test on the mean difference in error between the first guess and the average guess in the immediate condition vs. the delayed condition. As discussed in the Introduction, Vul and Pashler (2008) found that the benefit of averaging was greater in the delayed condition than in the immediate condition.

An executable Matlab script of the analyses can be found in Appendix A in the Supplementary Material.

## 3. Results

The raw data and post-processed data are available at the Open Science Framework (osf.io/ivfu6; Spies et al., 2012), accompanied with three Matlab scripts to execute the post-processing of the raw data, the pre-registered confirmatory analyses (see also Appendix B in the Supplementary Material)^6^ and additional *post-hoc* analyses. We adopted a co-pilot approach (Wicherts, 2011) in the sense that aside from the analyses based on this Matlab code, the second author independently post-processed and analyzed the data in SPSS, except for the calculation of the confidence intervals for the effect sizes and the calculation of the Bayes factors. The results obtained from these SPSS analyses were identical to the results from the Matlab analyses.

### 3.1. Sample

#### 3.1.1. Immediate condition

A total of 484 psychology students participated in the immediate condition. However, 11 of these participants did not complete the experiment, so the data from these participants were excluded in the data analysis. Following our pre-registered data cleaning plan, we also excluded the data from two participants who indicated that they had looked up the answers to the questions. Further, we were planning to exclude data from those participants who defocused the browser window while running the study. Yet, due to a technical problem, the digital assessment of whether participants had defocused the browser window in the immediate condition was not reliable. Fortunately, this is not problematic in this condition, as at the time of the data collection an experimenter was present in the back of the room, ascertaining that participants did not defocus the browser window. Finally, we were planning to exclude data when impossible answers (i.e., percentages below zero or above hundred) or blank answers were given. However, as it was made impossible to provide these type of answers in the experiment, this part of the data cleaning plan did not need to be executed. Our final sample of 471 psychology students consisted of 397 women and 74 men, with a mean age of 19.2 (*SD* = 2.8). Note that the gender imbalance in our sample is according to our expectations.

#### 3.1.2. Delayed condition

A total of 231 psychology students participated in the first session of the delayed condition and 171 of these students also participated in the second session. We excluded the data from 9 participants who did not complete one or both sessions, the data from 21 participants who defocused the browser window while running the study and the data from one participant who indicated that she had looked up the answers to the questions. Similar to the immediate condition, it was made impossible for participants to provide impossible or blank answers, so we did not need to exclude data based on these criteria. Our final sample of 140 participants consisted of 125 women and 15 men, with a mean age of 22.0 (*SD* = 3.1). Again, this gender imbalance is according to our expectations.

### 3.2. Confirmatory analysis

**Figure 1:**
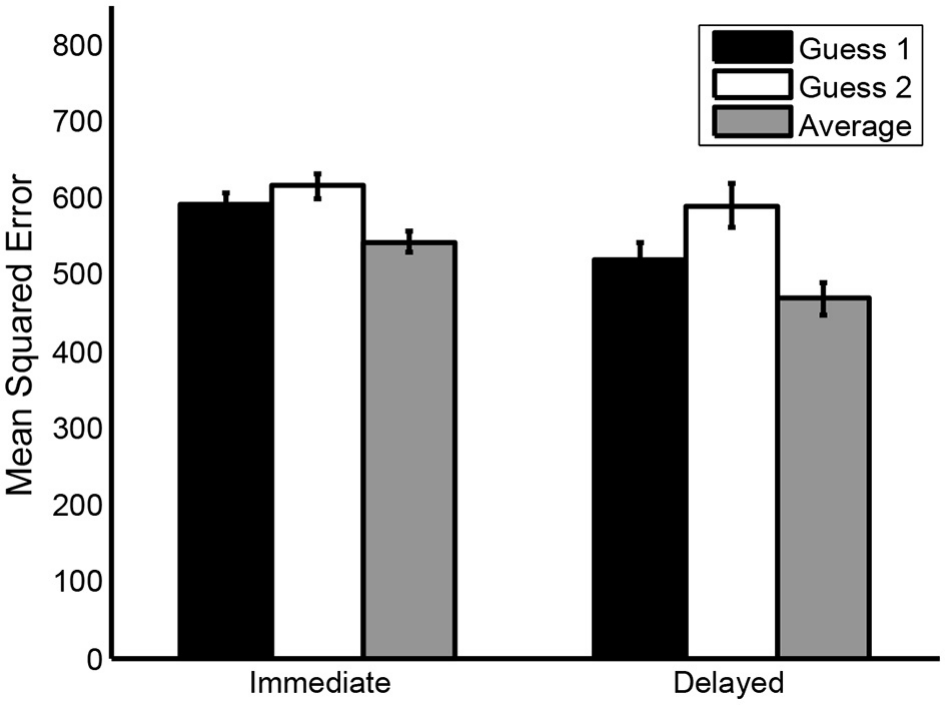
**Mean mean squared errors (MSE's) of guess 1, guess 2 and the average of both guesses in the immediate condition and the delayed condition**. Error bars represent standard errors.

As shown in Figure 1, both in the immediate and in the delayed condition, the accuracy of the aggregated guess was higher compared to the accuracy of the individual guesses (see also Table 4). In the immediate condition, the mean MSE of the average of the two guesses (*M* = 541, *SD* = 313) was smaller than both the mean MSE of guess 1 (*M* = 589, *SD* = 336), *t*(470) = 8.69, *p* < 0.001, ${\hat{d}}_{z}$ = 0.40, 95% CI = [0.31, 0.49] and the mean MSE of guess 2 (*M* = 615, *SD* = 351), *t*(470) = 10.26, *p* < 0.001, ${\hat{d}}_{z}$ = 0.47, 95% CI = [0.38, 0.57]. Likewise, in the delayed condition, the mean MSE of the average of the two guesses (*M* = 467, *SD* = 260) was smaller than both the mean MSE of guess 1 (*M* = 517, *SD* = 288), *t*(139) = 4.02, *p* < 0.001, ${\hat{d}}_{z}$ = 0.34, 95% CI = [0.17, 0.51] and the mean MSE of guess 2 (*M* = 589, *SD* = 327), *t*(139) = 8.48, *p* < 0.001, ${\hat{d}}_{z}$ = 0.72, 95% CI = [0.53, 0.90]. Thus, our results are comparable to the results obtained by Vul and Pashler (2008).

**Table 4:** **Statistics for guess 1 and guess 2 in the immediate condition and the delayed condition in the current study**.

**Condition**	**Mean MSE**	**Mean MSE**	***SD* MSE**	***SD* MSE**	***r***	***n***	***t***	***p***	**${\hat{d}}_{z}$**
	**single guess**	**average guess**	**single guess**	**average guess**					
**GUESS 1**									
Immediate condition	589	541	336	313	0.93	471	8.69	<0.001	0.40
Delayed condition	517	467	288	260	0.86	140	4.02	<0.001	0.34
**GUESS 2**									
Immediate condition	615	541	351	313	0.90	471	10.26	<0.001	0.47
Delayed condition	589	467	327	260	0.86	140	8.48	<0.001	0.72

*MSE = mean squared error; SD = standard deviation; r = correlation between single guess and average guess; n = sample size; t = dependent t-statistic value; p = p-value of corresponding statistic; ${\hat{d}}_{z}$ = effect size*.

According to the traditional standards for evaluating replication attempts, the current study can be considered as a successful replication of the crowd within effect, in both the immediate and the delayed condition. Another strategy for evaluating replication attempts has recently been proposed by Simonsohn (2013), who suggests to compare confidence intervals for effect sizes with the small effect size *d*_33%_, associated with a power of 33% in the original study. According to this *detectability* approach, a replication attempt is successful when the null hypothesis is rejected and the effect size estimate is not significantly smaller than *d*_33%_. Using G^*^Power 3.1, we calculated that *d*_33%_ = 0.10 in the immediate condition and *d*_33%_ = 0.12 in the delayed condition, based on the sample sizes from Vul and Pashler (2008). Clearly, our effect size estimates of both guesses in both conditions are larger than *d*_33%_, so against this criterion also, the current study is a successful replication of the crowd within effect in both the immediate and the delayed condition.

**Figure 2:**
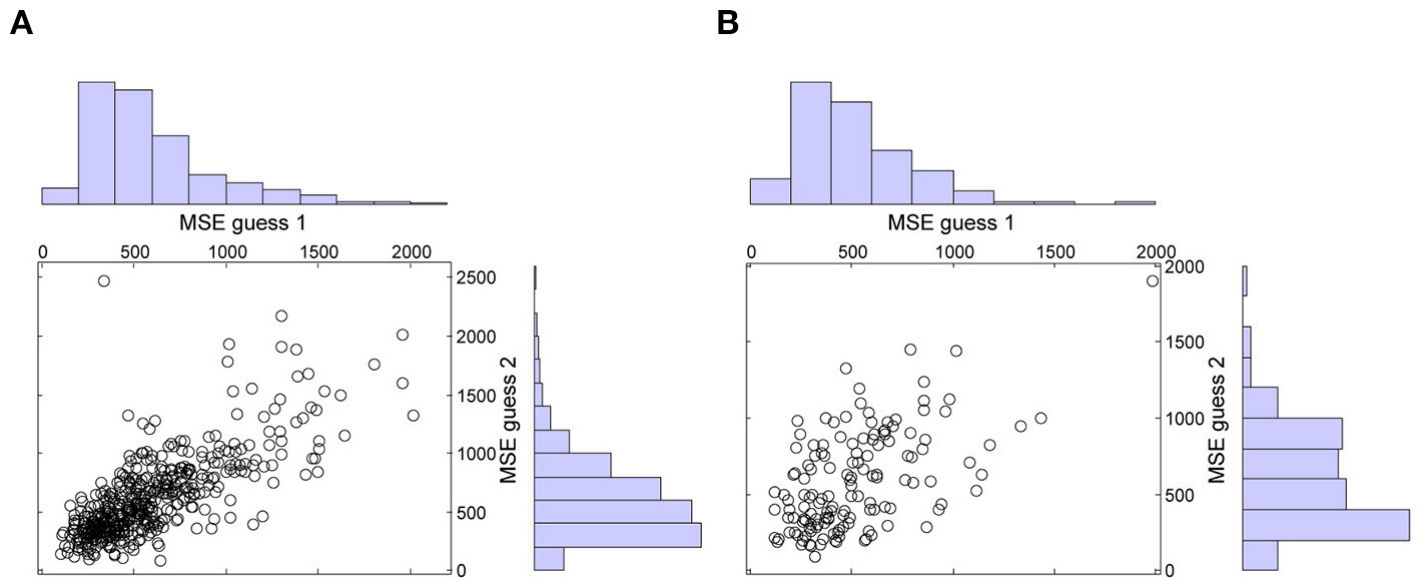
**Mean squared errors (MSE's) of guess 1 and guess 2 in the immediate **(A)** and in the delayed **(B)** condition**.

Following Vul and Pashler (2008), we performed two additional analyses. First, both in the immediate and in the delayed condition, second guesses were less accurate than first guesses. In the immediate condition, the mean MSE of guess 1 (*M* = 589, *SD* = 336) was smaller than the mean MSE of guess 2 (*M* = 615, *SD* = 351), *t*(470) = −2.25, *p* = 0.025, ${\hat{d}}_{z}$ = −0.10, 95% CI = [−0.19, −0.01]. Likewise, in the delayed condition, the mean MSE of guess 1 (*M* = 517, *SD* = 288) was smaller than the mean MSE of guess 2 (*M* = 589, *SD* = 327), *t*(139) = −2.91, *p* = 0.004, ${\hat{d}}_{z}$ = −0.25, 95% CI = [−0.41, −0.08]. These results reassure that the accuracy gain of averaging could not be attributed to participants looking up the answers between guesses. This is also confirmed by the scatter plots with the marginal histograms of the MSE's of guess 1 and guess 2 in both conditions (see Figure 2). As noted by Vul (n.d.), if participants were looking up the answers, there should be a peak in the error histograms at the value that can be expected when people know the right answer, i.e., error = 0. Clearly, this is not the case in Figure 2.

Second, unlike in Vul and Pashler (2008), the accuracy gain of averaging both guesses compared to guess 1 was not significantly larger in the delayed condition than in the immediate condition^7^. The mean difference between the MSE of the average and the MSE of guess 1 was not significantly larger in the delayed condition (*M* = 50, *SD* = 147) than in the immediate condition (*M* = 48, *SD* = 119), *t*(609) = 0.18, *p* = 0.858, $\hat{d}$ = 0.02, 95% CI = [−0.17, 0.21].

### 3.3. *Post-hoc* analyses

Since we were surprised by the non-significant difference between the immediate and the delayed condition in the accuracy gain of averaging guesses compared to guess 1, we also tested the difference between both conditions by comparing the average guess to guess 2. Unlike our comparison with guess 1, the mean difference between the MSE of the average and the MSE of guess 2 was significantly larger in the delayed condition (*M* = 121, *SD* = 169) than in the immediate condition (*M* = 73, *SD* = 155), *t*(609) = 3.14, *p* = 0.002, $\hat{d}$ = 0.30, 95% CI = [0.11, 0.49]. As this comparison was not reported in the original paper, we performed the same analysis on the raw data of the original study, which were provided to us by Edward Vul. Unlike in the current study, the mean difference between the MSE of the average and the MSE of guess 2 was not significantly larger in the delayed condition (*M* = 164, *SD* = 218) than in the immediate condition (*M* = 131, *SD* = 211), *t*(426) = 1.56, *p* = 0.12, $\hat{d}$ = 0.15.

In sum, the evidence for the difference in magnitude of the crowd within effect between the immediate condition and the delayed condition is mixed. Whereas the original study by Vul and Pashler (2008) yields a significant difference between both conditions when the average guess is compared to guess 1, but not when it is compared to guess 2, the present study yields the opposite pattern: When the average guess is compared to guess 2, there is a significant difference between both conditions, but when it is compared to guess 1, the difference is not significant. Thus, in both studies, introducing a three-week delay increased the benefit of averaging compared to one of both guesses only. However, it is important to keep in mind the exploratory nature of these analyses, as we did not use a power analysis to determine the sample size for testing these effects and we did not a priori specify the comparison of the average with guess 2.

### 3.4. Bayes factors for confirmatory and *post-hoc* analyses

The Bayesian alternative to null hypothesis significance testing is calculating the Bayes factor (BF), which quantifies the evidence of the null hypothesis relative to the alternative hypothesis. In contrast to a *p*-value, the BF can provide evidence both in favor or against the null hypothesis. Therefore, in addition to *p*-values, we calculated BF's for all tests, using a web-based Bayes factor calculator^8^. Table 5 shows the BF's for all tests of the confirmatory and *post-hoc* analyses, together with the *t*-statistics, sample sizes and *p*-values. All BF's show qualitatively identical results as the null hypothesis significance tests (i.e., tests with *p* < 0.05 have BF < 1, indicating evidence for the alternative hypothesis, whereas tests with *p* > 0.05 have *BF* > 1, indicating evidence for the null hypothesis), except for the comparison between the error of guess 1 and guess 2 in the immediate condition. The *p*-value for this latter test is 0.025, suggesting the difference is significant, whereas the BF is 2.208, indicating anecdotal evidence for the null hypothesis of no difference. However, both results are consistent with participants not having looked up the answers between guesses.

**Table 5:** **JZS Bayes factors (BF, with scale *r* = 1) in favor of the null hypothesis of no difference for all tests**.

***t***	***n***	***p***	***BF***
8.69	471	<0.001	1.8 10^−14^
10.26	471	<0.001	7.1 10^−20^
4.02	140	<0.001	0.007
8.48	140	<0.001	4.7 10^−12^
−2.25	471	0.025	2.208
−2.91	140	0.004	0.252
0.18	471 (*n*_1_) & 140 (*n*_2_)	0.858	12.931
3.14	471 (*n*_1_) & 140 (*n*_2_)	0.002	0.107

*The first seven tests are from the confirmatory analyses, the last test is from the post-hoc analyses*.

## 4. Discussion

Our replication attempt of the crowd within effect supports the original finding by Vul and Pashler (2008) that averaging two guesses within one person provides a more accurate answer than either guess alone. This effect was found when the second guess was made immediately after the first guess (immediate condition), as well as when the second guess was made 3 weeks later (delayed condition). These results were evaluated as successful replications against two different replication evaluation standards: The traditional *p*-value approach on the one hand, and the recently proposed detectability approach on the other hand.

The three-week delay between the two guesses improved the accuracy gain of averaging compared to guess 2, but not compared to guess 1. These results are comparable to those in Vul and Pashler (2008), where an increase in accuracy gain was also observed with the comparison of one of both guesses only. Thus, it seems that more research is needed to investigate whether a temporal separation between guesses can boost the crowd within effect.

### Conflict of interest statement

The authors declare that the research was conducted in the absence of any commercial or financial relationships that could be construed as a potential conflict of interest.

## Footnotes

^1^In Hourihan and Benjamin (2010), a minority of the subjects made the second guess after a delay of less than an hour instead of immediately following the first guess. However, Hourihan and Benjamin (2010) reported that this procedural difference did not affect the data, so it was not examined further.

^2^More specifically, the reported accuracy gain (defined as the median decrease in error of the average of the two estimates relative to the first estimate, across items) was on average 0.3% points with *SD* = 2.3%, Mdn = 0%, CI = [0.0 − 0.8 %] and *d* = 0.12.

^3^Whereas Vul and Pashler (2008) measured accuracy gain by taking the difference between the mean squared error (MSE) of one of the single estimates and the MSE of the aggregated guess, Herzog and Hertwig (2009) defined accuracy gain as the median decrease in absolute error of the aggregated estimates relative to the first estimate.

^4^We planned that in the unlikely case that the planned sample size was not reached (i.e., there were less than 48 students participating twice), we would repeat the procedure in another course and combine the data from both courses in our analyses. However, this turned out to be unnecessary.

^5^We retrieved these questions from Edward Vul's webpage (Vul, n.d.).

^6^Whereas Appendix A in the Supplementary Material contains the pre-registered Matlab code for the confirmatory analyses, we used the code in Appendix B in the Supplementary Material. The code in Appendix B in the Supplementary Material extends the code in Appendix A in the Supplementary Material in that it contains code for the calculation of descriptive statistics, some effect sizes, some confidence intervals for effect sizes, and code to make the scatter histogram plots in Figure 2.

^7^Contacting the authors concerning the experiment led them to uncover that their data show a slightly stronger evidence for this accuracy gain effect between both conditions than what had been reported in the article originally. The reported *t*-statistic, [*t*(426) = 2.12, *p* < 0.05], was smaller than the correct *t*-value, [*t*(426) = 2.68, *p* = 0.008].

^8^The Bayes factor calculator, available at http://pcl.missouri.edu/bayesfactor, requires input of the *t*-statistic and the sample size only. Following Rouder et al. (2009)'s recommendations, we report the JZS Bayes factor.
