# Corrigendum: Measuring the crowd within again: a pre-registered replication study

**DOI:** 10.3389/fpsyg.2015.00238

**Published:** 2015-03-12

**Authors:** Sara Steegen, Laura Dewitte, Francis Tuerlinckx, Wolf Vanpaemel

**Affiliations:** Research Group of Quantitative Psychology and Individual Differences, Faculty of Psychology and Educational Sciences, University of LeuvenLeuven, Belgium

**Keywords:** crowd within, registered replication study, power analysis, wisdom of the crowd, effect size

This is a corrigendum for Measuring the crowd within again: a pre-registered replication study.

The target sample sizes for achieving a 0.95 power level reported in the Sampling Plan section are incorrect. Specifically, the target sample sizes in the immediate condition should be *n* = 452 and *n* = 44 (instead of the reported *n* = 439 and *n* = 31); the target sample sizes in the delayed condition should be *n* = 61 and *n* = 26 (instead of the reported *n* = 48 and *n* = 13).

As the effective samples sizes, as reported in the Sample section (*n* = 471 in the immediate condition; *n* = 140 in the delayed condition), exceed the corrected target sample sizes in both conditions, the errors are non-consequential.

Additional Matlab code for the sampling plan can be found on the Open Science Framework (osf.io/ivfu6).

## Conflict of interest statement

The authors declare that the research was conducted in the absence of any commercial or financial relationships that could be construed as a potential conflict of interest.

