# Supplementary material for: Measuring the crowd within again: a pre-registered replication study
Source: Front Psychol. 2014 Jul 28;5:786. doi: 10.3389/fpsyg.2014.00786 (PMC4112915; doi:10.3389/fpsyg.2014.00786)
Supplement: Supplementary file 1 [file Presentation1.PDF]

Appendix A

Pre-registered Matlab Script for Confirmatory Analyses

```
% This code is based on code written by Edward Vul,
% except for the computation of the confidence intervals for the
% effect sizes, which is based on code provided by Simonsohn  (2013).
data;
% The data are 2 nx16 matrices (the first matrix with data from
% immediate condition; the second matrix with data from the the ...
    delayed
% condition), where n is the sample size of the corresponding ...
    condition.
% Each row corresponds to the answers from one participant. The ...
    first 8
% columns correspond to the first guesses; the final 8 colums ...
    correspond to
% the second guesses.

answers = [6.3 43.3 32.3 13.4 53.6 54.8 26.4 22.4];
% These answers are derived from The World Factbook (Central ...
    Intelligence
% Agency, 2013)

%% sets are 1: immediate, 2: delayed

for set = [1:2]
    [si sj] = size(data{set});
    grp(set).n=si;
    % guess 1
    grp(set).g{1} = data{set}(:,[1:8]);
    % guess 2
```

```
    grp(set).g{2} = data{set}(:,[9:16]);
    % average of guesess
    grp(set).g{3} = (grp(set).g{1}+grp(set).g{2})./2;

    for g = [1:3]
        %[n k] = size(grp(set).g{1});
        bigans = repmat(answers, grp(set).n, 1);
        % mean squared error
        grp(set).d{g} = mean((grp(set).g{g}-bigans).^2,2);
        grp(set).mumse(g) = mean(grp(set).d{g});
        grp(set).semse(g) = std(grp(set).d{g})./sqrt(grp(set).n);
    end
end

%% graph of mean MSE guess 1, guess2, average guess

figure();
barweb([grp(1).mumse; grp(2).mumse], [grp(1).semse; grp(2).semse]);
ylim([400 700]);

%% comparisons between guess and average

for set = [1:2]
    for g = [1:2]
    % guess 1 or guess 2 compared to average.
    % ttest
    [h p ci stats] = ttest(grp(set).d{g} - grp(set).d{3});
    grp(set).t{g} = stats.tstat;
    grp(set).p{g} = p;
    % effect size
    grp(set).dz{g} = grp(set).t{g}/sqrt(grp(set).n);
    % confidence interval (see Cumming and Finch (2001, pp. 544-545))
    alpha=.05;
    df = grp(set).n-1;
```

```
58      tnonct = inline('nctcdf(x,df,delta) - pr');
59      ncp_low = fzero(@(delta) tnonct(delta, df, 1-alpha/2, ...
            grp(set).t{g}), [-20,20]);
60      ncp_high = fzero(@(delta) tnonct(delta, df, alpha/2, ...
            grp(set).t{g}), [-20,20]);
61      grp(set).dzlow{g} = ncp_low/sqrt(grp(set).n);
62      grp(set).dzhigh{g} = ncp_high/sqrt(grp(set).n);
63      end
64
65      % guess 1 compared to guess 2.
66      [h3 p3 ci3 stats3] = ttest(grp(set).d{1} - grp(set).d{2});
67  end
68
69
70  %% comparison between magnitude of averaging benefit over guess 1.
71  [h4 p4 ci4 stats4] = ttest2(grp(2).d{1} - grp(2).d{3}, ...
        grp(1).d{1} - grp(1).d{3});
```

Appendix B

Used Matlab Script for Confirmatory Analyses

```
% This is the code used for the confirmatory analyses.
% Apart from minor syntactic clean-up, this code differs from the
% pre-registered code in Appendix A in that we added code for
% 1) descriptive statistics
% 2) the scatter histogram plots in Figure 2
% 3) some effect sizes
% 4) some confidence intervals for effect sizes.
% Further, the appearance of the bar chart (Figure 1) is changed.

% The data file (data_steegenetal2014.mat) is made from several ...
    .txt files,
% using the code from steegenetal2014_postprocessing.m

% This code is based on code written by Ed Vul,
% except for the computation of the confidence intervals for the
% effect sizes, which is based on code provided by Simonsohn  (2013).
clear all
close all
load data_steegenetal2014;
% The data are 2 nx16 matrices (the first matrix with data from
% immediate condition; the second matrix with data from the the ...
    delayed
% condition), where n is the sample size of the corresponding ...
    condition.
% Each row corresponds to the answers from one participant. The ...
    first 8
% columns correspond to the first guesses; the final 8 colums ...
    correspond to
% the second guesses.
```

```
25
26 % The code relies on barweb.m, which is available on matlabcentral
27
28 % Steegen, S., Dewitte, L., Tuerlinckx, F., & Vanpaemel, W. ...
       (2014). Measuring the crowd within again: A pre-registered ...
       replication study. Frontiers in Psychology
29
30 answers = [6.3 43.3 32.3 13.4 53.6 54.8 26.4 22.4];
31 % These answers are derived from The World Factbook (Central ...
       Intelligence
32 % Agency, 2013)
33
34 % answers = [6.3 44.4 30.3 10.5 58 72.4 18.9 20.3]; these are the ...
       answers
35 % used in Vul & Pashler (2008) (see first colomn Table 3)
36
37 %%%%%%%%%%%%%%%%%%%%%%%%%%%%%%%%%%%%%%%%%%%%%%%%%%%%%%%%%%%%%%%
38 % compute MSE, descriptive statistics and plot data
39 %%%%%%%%%%%%%%%%%%%%%%%%%%%%%%%%%%%%%%%%%%%%%%%%%%%%%%%%%%%%%%%
40
41 %% sets are 1: immediate, 2: delayed
42
43 for set = [1:2]
44     [si sj] = size(data{set});
45     grp(set).n=si;
46     % guess 1
47     grp(set).g{1} = data{set}(:,[1:8]);
48     % guess 2
49     grp(set).g{2} = data{set}(:,[9:16]);
50     % average of guesses
51     grp(set).g{3} = (grp(set).g{1}+grp(set).g{2})./2;
52
53     for g = [1:3]
54         bigans = repmat(answers, grp(set).n, 1);
```

```
55          % mean squared error
56          grp(set).d{g} = nanmean((grp(set).g{g}-bigans).^2,2);
57          grp(set).mumse(g) = nanmean(grp(set).d{g});
58          grp(set).semse(g) = nanstd(grp(set).d{g})./sqrt(grp(set).n);
59      end
60  end
61
62  % descriptive statistics
63  for set = [1:2]
64  grp(set).sdmse = grp(set).semse.*sqrt(grp(set).n);
65      for g=[1:2]
66          grp(set).corr{g}=corr(grp(set).d{g}, grp(set).d{3});
67          grp(set).mudiff{g} = mean(grp(set).d{g} - grp(set).d{3});
68          grp(set).sddiff{g} = std(grp(set).d{g} - grp(set).d{3});
69      end
70  end
71
72  %% graph of mean MSE guess 1, guess2, average guess
73  figure();
74  barweb([grp(1).mumse; grp(2).mumse], [grp(1).semse; ...
        grp(2).semse], [],{'Immediate'; 'Delayed '},[],[],'Mean ...
        Squared Error',[0 0 0; 1 1 1; .5 .5 .5],[],{'Guess 1'; 'Guess ...
        2'; 'Average'},'WestEast');
75  ylim([0 850]);
76  legend({'Guess 1'; 'Guess 2'; 'Average'},'Location','Northeast')
77
78  % scatter histogram plots
79  figure();
80  scatterhist(grp(1).d{1},grp(1).d{2}, 'Location', 'NorthEast', ...
        'Direction', 'Out', 'Color', 'k')
81  xlabel('MSE guess 1', 'FontSize', 12)
82  ylabel('MSE guess 2', 'FontSize', 12)
83  figure
```

```
scatterhist(grp(2).d{1}, grp(2).d{2}, 'Location', 'NorthEast', ...
    'Direction', 'Out', 'Color', 'k')
xlabel('MSE guess 1', 'FontSize', 12)
ylabel('MSE guess 2', 'FontSize', 12)


%%%%%%%%%%%%%%%%%%%
% inferential tests
%%%%%%%%%%%%%%%%%%%

alpha=.05;

%% comparisons between guess and average and between guess 1 and ...
    guess 2

for set = [1:2]
    % guess 1 or guess 2 compared to average.
    for g = [1:2]
    % ttest
    [h p ci stats] = ttest(grp(set).d{g} - grp(set).d{3});
    grp(set).t{g} = stats.tstat;
    grp(set).df{g} = stats.df;
    grp(set).p{g} = p;
    % effect size
    grp(set).dz{g} = grp(set).t{g}/sqrt(grp(set).n);
    % confidence interval (see Cumming and Finch (2001, pp. 549-550))
    df = grp(set).df{g};
    tnonct = inline('nctcdf(x,df,delta) - pr');
    ncp_low = fzero(@(delta) tnonct(delta, df, 1-alpha/2, ...
        grp(set).t{g}), [-20,20]);
    ncp_high = fzero(@(delta) tnonct(delta, df, alpha/2, ...
        grp(set).t{g}), [-20,20]);
    grp(set).dzlow{g} = ncp_low/sqrt(grp(set).n);
    grp(set).dzhigh{g} = ncp_high/sqrt(grp(set).n);
```

```
114     end
115
116     % guess 1 compared to guess 2.
117     % ttest
118     [h p ci stats] = ttest(grp(set).d{1} - grp(set).d{2});
119     grp(set).t{3} = stats.tstat;
120     grp(set).df{3} = stats.df;
121     grp(set).p{3} = p;
122     % effect size
123     grp(set).dz{3} = grp(set).t{3}/sqrt(grp(set).n);
124     % confidence interval
125     df = grp(set).df{3};
126     tnonct = inline('nctcdf(x,df,delta) - pr');
127     ncp_low = fzero(@(delta) tnonct(delta, df, 1-alpha/2, ...
            grp(set).t{3}), [-20,20]);
128     ncp_high = fzero(@(delta) tnonct(delta, df, alpha/2, ...
            grp(set).t{3}), [-20,20]);
129     grp(set).dzlow{3} = ncp_low/sqrt(grp(set).n);
130     grp(set).dzhigh{3} = ncp_high/sqrt(grp(set).n);
131 end
132
133
134 %% comparison of magnitude of averaging benefit over guess 1 ...
       between immediate and delayed condtion.
135 % ttest
136 [h p ci stats] = ttest2(grp(2).d{1} - grp(2).d{3}, grp(1).d{1} - ...
       grp(1).d{3});
137 grpcmp.t{1} = stats.tstat;
138 grpcmp.df{1} = stats.df;
139 grpcmp.p{1} = p;
140 % effect size (cohen's standardized mean difference d for ...
       independent groups; see Cumming and Finch (2001, pp. 567)
141 grpcmp.d{1} = grpcmp.t{1}.*sqrt((1/grp(1).n)+(1/grp(2).n));
142 % confidence interval (see Cumming and Finch (2001, pp. 567))
```

```
df = grpcmp.df{1};
tnonct = inline('nctcdf(x,df,delta) - pr');
ncp_low = fzero(@(delta) tnonct(delta, df, 1-alpha/2, ...
    grpcmp.t{1}), [-20,20]);
ncp_high = fzero(@(delta) tnonct(delta, df, alpha/2, ...
    grpcmp.t{1}), [-20,20]);
grpcmp.dlow{1} = ncp_low.*sqrt((1/grp(1).n)+(1/grp(2).n));
grpcmp.dhigh{1} = ncp_high.*sqrt((1/grp(1).n)+(1/grp(2).n));
```
